# Supplementary material for: Iterative improvement in the automatic modular design of robot swarms
Source: PeerJ Comput Sci. 2020 Dec 7;6:e322. doi: 10.7717/peerj-cs.322 (PMC7924708; doi:10.7717/peerj-cs.322)
Supplement: Supplemental Information 3 [file peerj-cs-06-322-s003.zip › argos3/doc/api/standalone/a00562_source.html]

ARGoS: plugins/robots/spiri/simulator/spiri\_entity.cpp Source File


- Main Page
- Related Pages
- Namespaces
- Classes
- Files

- File List
- File Members

# plugins/robots/spiri/simulator/spiri\_entity.cpp

Go to the documentation of this file.

```
00001 
00007 #include "spiri_entity.h"
00008 
00009 #include <argos3/core/simulator/space/space.h>
00010 #include <argos3/core/simulator/entity/controllable_entity.h>
00011 #include <argos3/core/simulator/entity/embodied_entity.h>
00012 #include <argos3/plugins/simulator/entities/led_equipped_entity.h>
00013 #include <argos3/plugins/simulator/entities/light_sensor_equipped_entity.h>
00014 #include <argos3/plugins/simulator/entities/perspective_camera_equipped_entity.h>
00015 #include <argos3/plugins/simulator/entities/proximity_sensor_equipped_entity.h>
00016 #include <argos3/plugins/simulator/entities/quadrotor_entity.h>
00017 #include <argos3/plugins/simulator/entities/rab_equipped_entity.h>
00018 
00019 namespace argos {
00020 
00021    /****************************************/
00022    /****************************************/
00023 
00024    static const Real HEIGHT         = 0.090f;
00025    static const Real BODY_HEIGHT    = HEIGHT * 3.0f / 4.0f;
00026    static const Real BODY_SIDE      = 0.470f;
00027    static const Real BODY_DIAGONAL  = BODY_SIDE * ::sqrt(2);
00028    static const Real BODY_RADIUS    = BODY_DIAGONAL / 2.0f;
00029    static const Real BODY_ELEVATION = HEIGHT - BODY_HEIGHT;
00030    static const Real RAB_ELEVATION  = (BODY_ELEVATION + BODY_HEIGHT) / 2.0f;
00031 
00032    /****************************************/
00033    /****************************************/
00034 
00035    CSpiriEntity::CSpiriEntity() :
00036       CComposableEntity(NULL),
00037       m_pcControllableEntity(NULL),
00038       m_pcEmbodiedEntity(NULL),
00039       m_pcQuadRotorEntity(NULL),
00040       m_pcRABEquippedEntity(NULL),
00041       m_pcPerspectiveCameraEquippedEntity(NULL) {
00042    }
00043 
00044    /****************************************/
00045    /****************************************/
00046 
00047    CSpiriEntity::CSpiriEntity(const std::string& str_id,
00048                               const std::string& str_controller_id,
00049                               const CVector3& c_position,
00050                               const CQuaternion& c_orientation,
00051                               Real f_rab_range,
00052                               size_t un_rab_data_size,
00053                               const CRadians& c_cam_aperture,
00054                               Real f_cam_range) :
00055       CComposableEntity(NULL, str_id),
00056       m_pcControllableEntity(NULL),
00057       m_pcEmbodiedEntity(NULL),
00058       m_pcQuadRotorEntity(NULL),
00059       m_pcRABEquippedEntity(NULL),
00060       m_pcPerspectiveCameraEquippedEntity(NULL) {
00061       try {
00062          /*
00063           * Create and init components
00064           */
00065          /*
00066           * Embodied entity
00067           * Better to put this first, because many other entities need this one
00068           */
00069          m_pcEmbodiedEntity = new CEmbodiedEntity(this, "body_0", c_position, c_orientation);
00070          AddComponent(*m_pcEmbodiedEntity);
00071          /* Quadrotor entity */
00072          m_pcQuadRotorEntity = new CQuadRotorEntity(this, "quadrotor_0");
00073          AddComponent(*m_pcQuadRotorEntity);
00074          /* RAB equipped entity */
00075          SAnchor& cRABAnchor = m_pcEmbodiedEntity->AddAnchor(
00076             "rab",
00077             CVector3(0.0f, 0.0f, RAB_ELEVATION));
00078          m_pcRABEquippedEntity = new CRABEquippedEntity(
00079             this,
00080             "rab_0",
00081             un_rab_data_size,
00082             f_rab_range,
00083             cRABAnchor,
00084             *m_pcEmbodiedEntity);
00085          AddComponent(*m_pcRABEquippedEntity);
00086          m_pcEmbodiedEntity->EnableAnchor("rab");
00087          /* Perspective camera */
00088          SAnchor& cCameraAnchor =
00089             m_pcEmbodiedEntity->AddAnchor(
00090                "camera",
00091                CVector3(BODY_RADIUS, 0.0f, 0.0f));
00092          m_pcPerspectiveCameraEquippedEntity = new CPerspectiveCameraEquippedEntity(
00093             this,
00094             "perspective_camera_0",
00095             c_cam_aperture,
00096             0.035f,
00097             f_cam_range,
00098             640, 480,
00099             cCameraAnchor);
00100          AddComponent(*m_pcPerspectiveCameraEquippedEntity);
00101          m_pcEmbodiedEntity->EnableAnchor("camera");
00102          /* Controllable entity
00103             It must be the last one, for actuators/sensors to link to composing entities correctly */
00104          m_pcControllableEntity = new CControllableEntity(this, "controller_0");
00105          AddComponent(*m_pcControllableEntity);
00106          m_pcControllableEntity->SetController(str_controller_id);
00107          /* Update components */
00108          UpdateComponents();
00109       }
00110       catch(CARGoSException& ex) {
00111          THROW_ARGOSEXCEPTION_NESTED("Failed to initialize entity \"" << GetId() << "\".", ex);
00112       }
00113    }
00114 
00115    /****************************************/
00116    /****************************************/
00117 
00118    void CSpiriEntity::Init(TConfigurationNode& t_tree) {
00119       try {
00120          /*
00121           * Init parent
00122           */
00123          CComposableEntity::Init(t_tree);
00124          /*
00125           * Create and init components
00126           */
00127          /*
00128           * Embodied entity
00129           * Better to put this first, because many other entities need this one
00130           */
00131          m_pcEmbodiedEntity = new CEmbodiedEntity(this);
00132          AddComponent(*m_pcEmbodiedEntity);
00133          m_pcEmbodiedEntity->Init(GetNode(t_tree, "body"));
00134          /* Quadrotor entity */
00135          m_pcQuadRotorEntity = new CQuadRotorEntity(this, "quadrotor_0");
00136          AddComponent(*m_pcQuadRotorEntity);
00137          /* RAB equipped entity */
00138          Real fRABRange = 3.0f;
00139          GetNodeAttributeOrDefault(t_tree, "rab_range", fRABRange, fRABRange);
00140          UInt32 unRABDataSize = 10;
00141          GetNodeAttributeOrDefault(t_tree, "rab_data_size", unRABDataSize, unRABDataSize);
00142          SAnchor& cRABAnchor = m_pcEmbodiedEntity->AddAnchor("rab", CVector3(0.0f, 0.0f, RAB_ELEVATION));
00143          m_pcRABEquippedEntity = new CRABEquippedEntity(
00144             this,
00145             "rab_0",
00146             unRABDataSize,
00147             fRABRange,
00148             cRABAnchor,
00149             *m_pcEmbodiedEntity);
00150          AddComponent(*m_pcRABEquippedEntity);
00151          m_pcEmbodiedEntity->EnableAnchor("rab");
00152          /* Perspective camera */
00153          CDegrees cCameraAperture(30.0f);
00154          GetNodeAttributeOrDefault(t_tree, "camera_aperture", cCameraAperture, cCameraAperture);
00155          Real fCameraRange = 10.0f;
00156          GetNodeAttributeOrDefault(t_tree, "camera_range", fCameraRange, fCameraRange);
00157          SAnchor& cCameraAnchor =
00158             m_pcEmbodiedEntity->AddAnchor(
00159                "camera",
00160                CVector3(BODY_RADIUS, 0.0f, 0.0f));
00161          m_pcPerspectiveCameraEquippedEntity = new CPerspectiveCameraEquippedEntity(
00162             this,
00163             "perspective_camera_0",
00164             ToRadians(cCameraAperture),
00165             0.035f,
00166             fCameraRange,
00167             640, 480,
00168             cCameraAnchor);
00169          AddComponent(*m_pcPerspectiveCameraEquippedEntity);
00170          m_pcEmbodiedEntity->EnableAnchor("camera");
00171          /* Controllable entity
00172             It must be the last one, for actuators/sensors to link to composing entities correctly */
00173          m_pcControllableEntity = new CControllableEntity(this);
00174          AddComponent(*m_pcControllableEntity);
00175          m_pcControllableEntity->Init(GetNode(t_tree, "controller"));
00176          /* Update components */
00177          UpdateComponents();
00178       }
00179       catch(CARGoSException& ex) {
00180          THROW_ARGOSEXCEPTION_NESTED("Failed to initialize entity \"" << GetId() << "\".", ex);
00181       }
00182    }
00183 
00184    /****************************************/
00185    /****************************************/
00186 
00187    void CSpiriEntity::Reset() {
00188       /* Reset all components */
00189       CComposableEntity::Reset();
00190       /* Update components */
00191       UpdateComponents();
00192    }
00193 
00194    /****************************************/
00195    /****************************************/
00196 
00197    REGISTER_ENTITY(CSpiriEntity,
00198                    "spiri",
00199                    "Carlo Pinciroli [ilpincy@gmail.com]",
00200                    "1.0",
00201                    "The spiri robot, developed by Pleiades Robotics.",
00202                    "The spiri is a quad-rotor developed by Pleiades Robotics. It is a\n"
00203                    "fully autonomous robot with a rich set of sensors and actuators. For more\n"
00204                    "information, refer to the dedicated web page (http://www.pleaides.ca/).\n\n"
00205                    "REQUIRED XML CONFIGURATION\n\n"
00206                    "  <arena ...>\n"
00207                    "    ...\n"
00208                    "    <spiri id=\"eb0\">\n"
00209                    "      <body position=\"0.4,2.3,0.25\" orientation=\"45,0,0\" />\n"
00210                    "      <controller config=\"mycntrl\" />\n"
00211                    "    </spiri>\n"
00212                    "    ...\n"
00213                    "  </arena>\n\n"
00214                    "The 'id' attribute is necessary and must be unique among the entities. If two\n"
00215                    "entities share the same id, initialization aborts.\n"
00216                    "The 'body/position' attribute specifies the position of the bottom point of the\n"
00217                    "spiri in the arena. When the robot is untranslated and unrotated, the\n"
00218                    "bottom point is in the origin and it is defined as the middle point between\n"
00219                    "the two wheels on the XY plane and the lowest point of the robot on the Z\n"
00220                    "axis, that is the point where the wheels touch the floor. The attribute values\n"
00221                    "are in the X,Y,Z order.\n"
00222                    "The 'body/orientation' attribute specifies the orientation of the spiri. All\n"
00223                    "rotations are performed with respect to the bottom point. The order of the\n"
00224                    "angles is Z,Y,X, which means that the first number corresponds to the rotation\n"
00225                    "around the Z axis, the second around Y and the last around X. This reflects\n"
00226                    "the internal convention used in ARGoS, in which rotations are performed in\n"
00227                    "that order. Angles are expressed in degrees. When the robot is unrotated, it\n"
00228                    "is oriented along the X axis.\n"
00229                    "The 'controller/config' attribute is used to assign a controller to the\n"
00230                    "spiri. The value of the attribute must be set to the id of a previously\n"
00231                    "defined controller. Controllers are defined in the <controllers> XML subtree.\n\n"
00232                    "OPTIONAL XML CONFIGURATION\n\n"
00233                    "You can set the emission range of the range-and-bearing system. By default, a\n"
00234                    "message sent by an spiri can be received up to 3m. By using the 'rab_range'\n"
00235                    "attribute, you can change it to, i.e., 4m as follows:\n\n"
00236                    "  <arena ...>\n"
00237                    "    ...\n"
00238                    "    <spiri id=\"eb0\" rab_range=\"4\">\n"
00239                    "      <body position=\"0.4,2.3,0.25\" orientation=\"45,0,0\" />\n"
00240                    "      <controller config=\"mycntrl\" />\n"
00241                    "    </spiri>\n"
00242                    "    ...\n"
00243                    "  </arena>\n\n"
00244                    "You can also set the data sent at each time step through the range-and-bearing"
00245                    "system. By default, a message sent by a spiri is 10 bytes long. By using the"
00246                    "'rab_data_size' attribute, you can change it to, i.e., 20 bytes as follows:\n\n"
00247                    "  <arena ...>\n"
00248                    "    ...\n"
00249                    "    <spiri id=\"eb0\" rab_data_size=\"20\">\n"
00250                    "      <body position=\"0.4,2.3,0.25\" orientation=\"45,0,0\" />\n"
00251                    "      <controller config=\"mycntrl\" />\n"
00252                    "    </spiri>\n"
00253                    "    ...\n"
00254                    "  </arena>\n\n"
00255                    ,
00256                    "Under development"
00257       );
00258 
00259    /****************************************/
00260    /****************************************/
00261 
00262    REGISTER_STANDARD_SPACE_OPERATIONS_ON_COMPOSABLE(CSpiriEntity);
00263 
00264    /****************************************/
00265    /****************************************/
00266 
00267 }
```

---

Generated on 10 Jul 2018 for ARGoS by 
 1.6.1
